# Cross-cultural validation of the COVID-19 peritraumatic distress index (CPDI) among Spanish and Peruvian populations

**DOI:** 10.1038/s41598-023-46235-4

**Published:** 2023-11-03

**Authors:** Fabian Böttcher, Bruno Pedraz-Petrozzi, Eva Kathrin Lamadé, Maria Pilar Jimenez, Jennifer Rieker, José Manuel Reales, Martin Arevalo-Flores, Víctor Anculle-Arauco, Hever Krüger-Malpartida, Soledad Ballesteros

**Affiliations:** 1 Department of Psychiatry and Psychotherapy, Central Institute of Mental Health, Medical Faculty Mannheim, University of Heidelberg, J5, 68159 Mannheim, Germany; 2 Departamento de Psicología Básica II, Universidad Nacional de Educación a Distancia, Calle Juan del Rosal 10, 28040 Madrid, Spain; 3 Facultad de Ciencias de la Vida y de la Naturaleza, Departamento de Psicología, Universidad de Nebrija, Calle de Santa Cruz de Marcenado, 27, 28015 Madrid, Spain; 4 Departamento de Metodología para las Ciencias Sociales, Universidad Nacional de Educación a Distancia, Calle Juan del Rosal 10, 28040 Madrid, Spain; 5 Facultad de Medicina, Alberto Hurtado”, Universidad Peruana Cayetano Heredia, Avenida Honorio Delgado 430, San Martín de Porres, 15102 Lima, Peru

**Keywords:** Human behaviour, Epidemiology, Psychology, Quality of life, Anxiety, Depression, Post-traumatic stress disorder, Risk factors

## Abstract

The COVID-19 pandemic has had a significant psychological impact worldwide. The COVID-19 Peritraumatic Distress Index (CPDI) is widely used to assess psychological stress during the COVID-19 pandemic. Although CPDI has been validated in Peru and Spain, no cross-cultural validation studies have been conducted. As an exploratory aim, differences in CPDI factorial scores between the most prevalent medical conditions in the two samples (arterial hypertension, respiratory diseases and anxious-depressive disorders) from a general population of Peru and Spain were investigated. We conducted secondary data analysis with data from Peru and Spain to validate the CPDI in a cross-cultural context. Exploratory factor analysis (EFA) and multigroup confirmatory factor analysis (MGCFA) were performed to evaluate the factor structure and measurement invariance of the CPDI across cultural contexts. Concerning the exploratory analysis, we performed a U-Mann–Whitney test to evaluate differences in the factorial scores in the two samples. This study revealed a two-factor solution (stress and rumination/information) for the CPDI that included 21 of the 24 original items, and consistent with previous studies. The MGCFA demonstrated measurement invariance across cultural contexts (scalar invariance), indicating that the CPDI construct has the same meaning across both groups, regardless of cultural context and language variations of Spanish. Patients with anxious-depressive disorders showed higher CPDI factorial scores for both factors, whereas patients with respiratory diseases were only associated with the stress factor. This study provides evidence for the cross-cultural validity of the CPDI, highlighting its utility as a reliable instrument for assessing psychological stress in the context of COVID-19 across different cultures. These findings have important implications for developing and validating measures to assess psychological distress in different cultural contexts.

## Introduction

The coronavirus disease 2019 (COVID-19) represented an epidemiological issue and a significant challenge that has negatively impacted the population, leading to changes in social behavior and individual lifestyles^1–3^. These negative changes, including lockdowns and social restrictions, have adversely affected the population's mental health, resulting in increased cases of depression^4^, trauma^5^, anxiety^6^, and suicidal behavior^7^. In response to this phenomenon, various research groups have developed different psychometric instruments to assess the negative effects of COVID-19 on the population, with some primarily focusing on distress during the COVID-19 lockdown. In 2019, Qiu and colleagues developed the COVID-19 Peritraumatic Distress Index (CPDI)^8^. This instrument is one of the pioneering tools to assess peritraumatic stress symptoms related to COVID-19. These symptoms include negative cognitive changes, avoidance, compulsive behavior, physical symptoms related to stress, social withdrawal, loss of social functioning, anxiety, and depressive symptoms. CPDI has been validated during the COVID-19 lockdown in different languages worldwide^9–12^, including European^11^ and Latin American Spanish^13,14^. Although both Spanish validation studies showed a two-factor solution, they showed variations in the items included in each factor and the interpretation of the factors. During the most critical period of the coronavirus pandemic, specific instruments have been used to assess coronavirus-related stress symptoms, such as the Fear Scale COVID-19 (FCV-19S)^15^, the Coronavirus Anxiety Scale (CAS)^16^, the COVID-19 Phobia Scale (C19 P-S)^17^, and the COVID-19 Peritraumatic distress Index (CPDI). Among these instruments, the CPDI probably captures the COVID-related psychological distress most comprehensively. As far as we know, cross-cultural validations in COVID-19 distress scales have been reported using the “fear of COVID-19 scale”^15^. Cross-cultural validations, assessed through measurement invariance, enable the study and validation of instrument results across different cultural groups, considering their differences^15,18^. Despite the frequent use of CPDI and the existence of different versions worldwide, a cross-cultural validation study using measurement invariance for the CPDI has been underreported.

### Objectives and hypotheses

For this purpose, the objective of this study was to evaluate the factorial structure and perform a cross-cultural validation using a measurement invariance analysis of the CPDI, using samples from the Spanish and Peruvian populations as examples, considering their cultural ties and shared language. As an exploratory aim, we investigated the differences in CPDI factorial scores between the three most prevalent medical conditions in both samples (i.e., arterial hypertension, respiratory diseases, and anxious-depressive disorders) after completing the cross-cultural validation. We expected that both samples would exhibit metric invariance, enabling comparisons between populations and providing valuable information about the utility of the CPDI in assessing peritraumatic distress experienced by individuals in Spanish and Peruvian populations. As part of our exploratory analysis, we expected that people with the above-mentioned medical conditions would have higher CPDI factorial scores in both samples than healthy subjects, in line with existing literature^19^.

## Materials and methods

### Participants and procedure

The present study is a secondary analysis of two databases comprising online survey studies conducted in Peru and Spain during the COVID-19 pandemic. Certain findings from this survey study have already been reported elsewhere^11,14^. The current study incorporates certain methodological aspects of both studies, primarily pertaining to the recruitment of participants, the description of the online survey, and the CPDI.

The primary objective of this study was to evaluate the psychometric properties of the Spanish version of the CPDI in two samples from Peru (N = 1469) and Spain (N = 1074) and to perform a cross-cultural validation using measurement invariance for both population samples (total N = 2543). As an exploratory objective, we evaluated the CPDI factorial score differences between the most common medical conditions among the two sample groups after performing the factorial and cross-cultural validation analysis.

The data for this study was originally collected from voluntary participants during the COVID-19 lockdown. The collection period for the Peru study was from March 27, 2020, to September 21, 2021, while for the Spain study, it was from May 8, 2020, to June 25, 2020.

In the Peruvian study, only residents during the COVID-19 lockdown aged 18 years or older, possessing sufficient Spanish language proficiency and providing written informed consent, were eligible for inclusion. Those who did not meet these criteria were excluded. Similarly, in the Spanish study, participants had to be Spanish residents aged 18 years or older. Both studies excluded individuals who did not fully complete the questionnaires or failed to provide socioeconomic information. Online electronic surveys were employed in both studies to collect participant information due to the sanitary restrictions in both countries, which prevented personal contact for data collection. In the Peruvian study, Google Forms, an open-access internet-based program provided by Google Inc, USA, was used for online surveys. For the Spanish study, the surveys were recorded automatically using the Qualtrics Software (Qualtrics Research Suite: Provo, UT, USA, 2013). Both surveys were conducted anonymously, and participants were only permitted to complete the survey once. The surveys included questions regarding socio-economic status (i.e., age, gender, education), psychometric data (CPDI scale) ^11,14^, and data of past medical history (i.e., actual medical conditions, current medications), extracting, in this case, the most frequent disorders in both populations (respiratory diseases, hypertension, hypercholesterinemia, diabetes mellitus, cardiovascular diseases, and anxious-depressive disorders) through direct questioning of the participants.

### COVID-19 peritraumatic distress index (CPDI)

The COVID-19 Peritraumatic Distress Index (CPDI) is a self-report questionnaire designed to assess psychological distress during the COVID-19 pandemic. It was first applied in China^8^. Regarding the psychometric properties of the CPDI the original version had an excellent internal consistency, Cronbach's α = 0.95^8^. Good reliability values were also found in a number of validation studies in different countries^9–12,20^.

This instrument comprises 24 items (e.g., “during this COVID-19 period, I often feel stomach pain, bloating, and other stomach discomfort”), each of which is evaluated on a Likert scale ranging from 0 to 4 (i.e., *never*, *occasionally*, *sometimes*, *often*, and *most of the time*). The raw score is calculated by adding the value of each item, and the displayed score is obtained by adding 4 to the raw score to determine the severity degree of CPDI. The CPDI defines different categories for peritraumatic stress related to the COVID-19 pandemic: *normal* (0–28 display points), *mild* (29–52 display points), and *severe* (53–100 display points) ^11,14,21^. In both studies, a validated version of the CPDI through expert adaptation and translation to the Spanish language was used, whose construct validation has been performed separately^11,14^.

### Data preparation and statistical analysis

Table representation and text description were used to present general sample characteristics, including descriptive data on the CPDI. For numerical variables that were normally distributed, means and standard deviation were used as measures of central tendency. For non-Gaussian distributed variables, median and interquartile ranges (IQR), including 75- and 25-percentiles, were used. Descriptive information greater than one million was expressed using scientific notation, and decimal data were rounded to two decimals. Qualitative data, including count data, was characterized using absolute numerical values and percentages. These procedures were performed for both subsamples, as shown in Table 1. Inferential statistical tests were not performed to evaluate differences between the two subsamples (i.e., first and second phase) since they corresponded to two sub projects with different objectives and hypotheses.

**Table 1:** Descriptive statistics of sociodemographic and health-related variables.

Variable	Level	Mean or n (SD or %)			t or χ^2^
		Total (n = 2543)	Spain (n = 1074)	Peru (n = 1469)	
Sex	Men	743 (29.2)	234 (21.8)	509 (34.6)	101.783***
	Women	1801 (70.8)	840 (78.2)	962 (65.4)	8.26**
Age		41.66 (15.47)	52.44 (14.10)	33.79 (13.31)	34.024***
Education	Primary education	58 (2.3)	57 (5.3)	1 (1.7)	54.069***
	Secondary education	419 (16.5)	239 (22.3)	180 (12.2)	8.308**
	Tertiary education/vocational training	262 (10.3)	134 (12.5)	128 (8.7)	0.137
	University or higher	1805 (71.0)	643 (59.9)	1162 (79.0)	149.23***
Diseases	Respiratory problems	227 (8.9)	84 (7.8)	143 (9.7)	2.735
	High cholesterol	182 (7.2)	170 (15.8)	12 (0.8)	210.941***
	Hypertension	233 (9.2)	168 (15.7)	65 (4.4)	94.183***
	Diabetes	69 (2.7)	38 (3.5)	31 (2.1)	4.836*
	Cardiovascular disease/s	38 (1.5)	22 (2.1)	16 (1.1)	3.907*
	Anxiety/depression	340 (13.4)	260 (24.2)	80 (5.4)	189.241***
Total CPDI		50.29 (14.78)	50.18 (15.32)	50.38 (14.38)	-0.353

*SD* standard deviation, *χ*^*2*^ Chi-square test, *CPDI* COVID-19 peritraumatic distress index.

Specifically, we investigated the factor structure of the CPDI in both populations. To achieve this, we merged the databases of both studies, randomly sorted the participants, and divided them into equal proportions (1:1 for each database) for exploratory factor analysis (EFA) and Multi-group confirmatory factor analysis (MGCFA), as recommended in previous studies^22^. In evaluating the EFA solution, we adhered to the following literature recommendations^23^:

- all factors should be theoretically meaningful
- at least three variables should saliently load on a factor (overdetermined, i.e., factor loadings ≥ 0.30)
- variables should load significantly on only one factor (no cross-loadings)
- each factor should have an internal consistency of Cronbach's α ≥ 0.70.

To determine whether cultural differences exist in the CPDI between the Spanish and Peruvian samples, it is essential to test the measurement invariance using MGCFA. This approach enables invariance evaluation by imposing cross-group restrictions and comparing models with varying degrees of constraints, as demonstrated in numerous studies ^24,25^. ANOVA was used to compare nested models that emerged by restricting different parameters and representing different levels of measurement invariance. The hypothesized measurement model's findings were evaluated using fit indices and their cut-off points: the comparative fit index (CFI), the Tucker-Lewis index (TLI), the root-mean-square-error of approximation (RMSEA), and the standardized root-mean-square-residual (SRMR) were used as model fit measurements. A good model fit was given if the values of the CFI and TLI were greater than or equal to 0.95^11^. Regarding RMSEA and SRMR, a good model fit was given if the values of both root-mean-square indicators were below or equal to 0.05^11^. Additionally, we calculated the 90-percent confidence intervals (90CI) for the RMSEA^26^. Differences in these alternative fit indices (AFI) were also used to compare the nested models, as the χ^2^-value is sensitive to sample size. In this case, following cutoffs were considered: −0.01 for ΔCFI, paired with 0.15 for ΔRMSEA and ΔSRMR of 0.030 (for metric invariance) or 0.015 (for scalar or residual invariance).

Finally, exploratory data analysis was conducted by calculating the sum of the scores of the resulting factors (i.e., factors 1 and 2) and assessing the differences in each factor score among the three most prevalent medical conditions in both samples.

Descriptive information was performed using JASP version 0.11.1^27^. Statistical analyses of the EFA were performed using the R-software version 4.1.2 (R Core Team, 2021, R Foundation for Statistical Computing, Vienna, Austria) ^28^ and for the MGCFA we utilized version 0.6-10 of the R-package lavaan^29^.

### Ethics approval and consent to participate

Before the commencement of data collection, participants in both studies were provided with complete information regarding the study and gave written informed consent. Each participant or their legally authorized representative was fully informed about the objectives and procedures of the study, as well as the potential adverse effects, and gave their written consent to participate. Both studies were conducted in accordance with the Helsinki Declaration. The study protocol and all study procedures were reviewed and approved by the Ethics Committee of the Universidad Peruana Cayetano Heredia (UPCH) for the Peruvian study and the Ethics Committee Board of the Universidad Nacional de Educación a Distancia (UNED) for the Spanish study. Additionally, this pilot trial was carried out according to the Helsinki Declaration.

## Results

### General descriptive data

The general characteristics of the Peruvian and Spanish samples, including socioeconomic variables and CPDI values, are presented in Table 1. Concerning medical conditions, most participants had anxious-depressive disorders (13.4%), arterial hypertension (9.2%), and respiratory diseases (8.9%). There were significant statistical differences in the frequency of medical conditions in Peruvian and Spanish samples (Table 1). Between both samples there were no significant differences concerning uncorrected CPDI values between the two samples.

### Exploratory factor analysis

For the EFA, the first subsample (*N* = 1271) was used, and we proceeded as follows: in the first step, the data was checked for adequacy considering the Kaiser–Meyer–Olkin (KMO)^30^ measure and the results of the Bartlett's sphericity test^31^. In the second step, the number of factors was determined using several extraction methods: parallel analysis^32^, Eigenvalues, visual Scree test^33^, and Velicer’s minimum average partial test (MAP)^34^. Finally, the EFA solution was evaluated considering the recommendations^23^ mentioned in the method section and adjustments were made if these were violated. The initial exploratory factor analysis (EFA) using Bartlett's sphericity test demonstrated that the correlation matrix results were not random (χ^2^_df = 276_ = 12,452.74, p < 0.001). Moreover, the KMO value indicated that the data was well-suited for factor analysis, with a KMO value of 0.94. The univariate skewness and kurtosis of the CPDI items were not extreme^35^ (see Table 2). Additionally the multivariate normal distribution of the items was estimated by Mardia's multivariate test for skewness and for kurtosis^36^. The null hypothesis for both tests was rejected (p < 0.001), suggesting a violation of multivariate normality of the CPDI in the first subsample. Due to the violation of standard distribution assumptions and the ordinal nature of the CPDI items, we used a polychoric correlation matrix as an input method for the EFA and a principal axis as a factor extraction method, in accordance with recommendations for the robustness of the principal axis method towards the violation of standard distribution assumptions as published elsewhere^37^.

**Table 2:** Factor loadings of principal axis analysis (promax-rotation) for CPDI and descriptive statistics for individual items.

Items	F1	F2	*M*	*SD*	Skewness	Kurtosis
Item 16: I find it hard to concentrate	0.88		2.62	1.26	0.40	−0.87
Item 14: I feel tired and sometimes even exhausted	0.85		2.46	1.24	0.50	−0.80
Item 15: Due to feelings of anxiety, my reactions are becoming sluggish	0.83		2.09	1.17	0.85	−0.24
Item 17: I find it hard to make any decisions	0.79		2.28	1.16	0.67	−0.37
Item 18: During this COVID-19 period, I often feel dizzy or have back pain and chest distress	0.78		1.92	1.14	1.12	0.28
Item 19: During this COVID-19 period, I often feel stomach pain, bloating, and other stomach discomfort	0.76		1.88	1.09	1.16	0.48
Item 1: Compared to usual, I feel more nervous and anxious	0.73		2.76	1.11	0.24	−0.66
Item 4: I feel empty and helpless no matter what I do	0.71		2.03	1.18	0.93	−0.16
Item 20: I feel uncomfortable when communicating with others	0.69		1.81	1.01	1.23	0.96
Item 13: I am more irritable and have frequent conflicts with my family	0.69		2.08	1.08	0.84	−0.05
Item 23: I lost my appetite	0.65		1.49	0.84	1.78	2.64
Item 24: I have constipation or frequent urination	0.58		1.73	1.07	1.43	1.15
Item 7: I am losing faith in the people around me	0.49		2.24	1.20	0.65	−0.54
Item 21: Recently, I rarely talk to my family	0.48		1.79	1.10	1.32	0.86
Item 12: I avoid watching COVID-19 news, since I am too scared to do so	0.42		2.10	1.21	0.93	−0.07
Item 11: I am constantly sharing news about COVID-19 (mostly negative news)		0.78	1.65	0.85	1.35	1.53
Item 8: I collect information about COVID-19 all day. Even if it's not necessary, I can not stop myself		0.77	2.34	1.13	0.68	−0.34
Item 9: I will believe the COVID-19 information from all sources without any evaluation		0.65	1.45	0.78	1.89	3.65
Item 10: I would rather believe in negative news about COVID-19 and be skeptical about the good news		0.60	1.83	0.99	1.12	0.79
Item 2: I feel insecure and bought a lot of masks, medications, sanitizer, gloves and/or other home supplies		0.44	2.08	1.07	0.81	−0.12
Item 6: I feel helpless and angry about people around me, governors, and media		0.34	3.10	1.20	0.01	−0.92

F1 = stress in the context of COVID-19 pandemics, F2 = rumination/information seeking in the context of COVID-19 pandemics.

**Figure 1:**
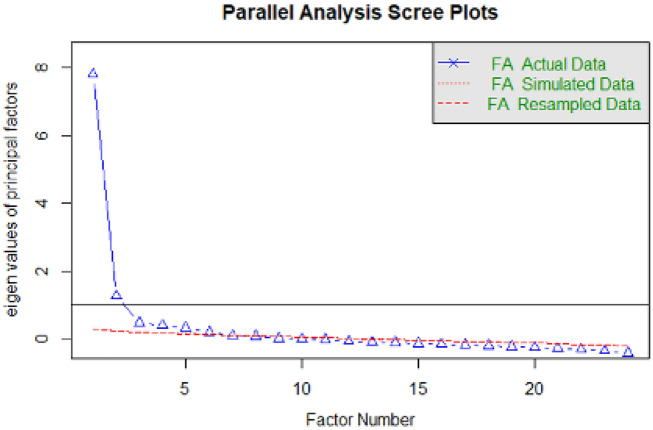
Parallel analysis scree plots for the exploratory factor analysis of the COVID-19 Peritraumatic distress index (CPDI) instrument in both Peruvian and Spanish sample sizes. Of note is the two-factor solution, which explains most of the variance for the CPDI.

All extraction methods indicated a two-factor solution. In addition, the parallel analysis scree plot for the EFA is represented in Fig. 1. We conducted an exploratory principal axis analysis with oblique rotation (promax) and deleted two items due to cross-loadings (item 22: λ_factor1_ = 0.35, λ_factor2_ = 0.40; item 3: λ_factor1_ = 0.34, λ_factor2_ = 0.44). With the remaining items, we ran another exploratory principal axis analysis, and the extraction methods used above were repeated, continuing to indicate a two-factor solution. We deleted item 5 due to low factor loading, λ = 0.29. The final 21 items were well-suited for factor analysis and could be well-assigned to the two factors. The two resulting factors are interpreted as follows: factor 1 or "*stress in the context of COVID-19 pandemics*" (e.g., "*I feel tired and sometimes even exhausted*"; Eigenvalue = 7.53, α = 0.91), and factor 2 or "*rumination/seeking for information in the context of COVID-19 pandemics*" (e.g., "*I can't stop myself from imagining myself or my family being infected and feel terrified and anxious about it*"; Eigenvalue = 2.41, α = 0.71). The resultant model accounted for 45% of the variation observed in the sample. Additionally, the two factors had a significant correlation (r = 0.53, p < 0.001). Table 2 shows factor loadings after oblique rotation and items statistics.

### Multi-group confirmatory factor analysis

**Figure 2:**
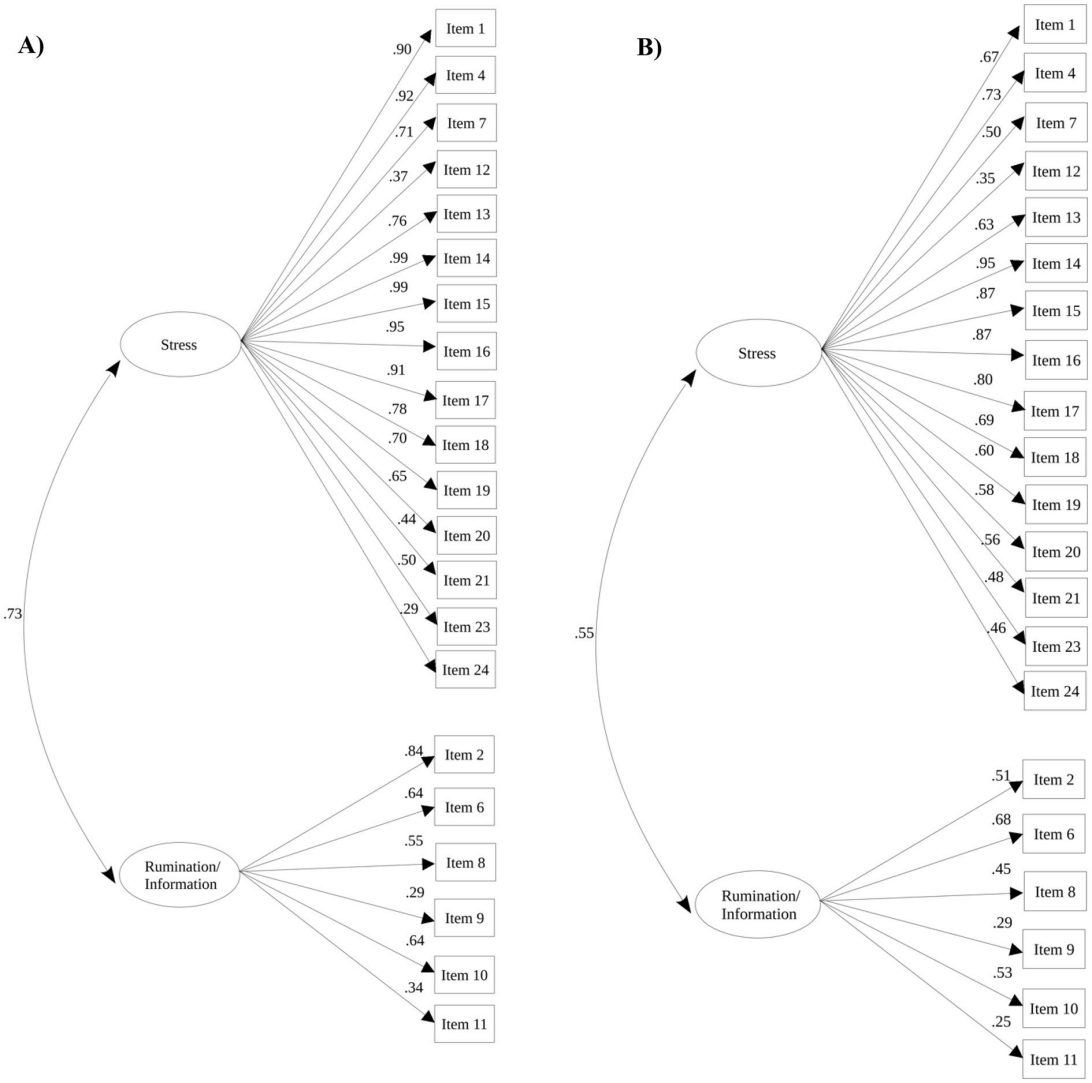
Multigroup confirmatory factor analysis, path diagram—standardized regression coefficients of the items on factor 1 (“stress in the context of COVID-19 pandemics”) and factor 2 (“rumination/information seeking in the context of COVID-19 pandemics”), for the Spanish (**A**) and Peruvian (**B**) sample sizes.

The aim of this study was to replicate the two-factor solution obtained in the exploratory factor analysis (EFA) in a second subsample (*N* = 1272) and examine metric invariance. To achieve this, a multi-group confirmatory factor analysis (MGCFA) was performed using the diagonal weighted least square (DWLS) estimation method. The hypothesized two-factor model had a good fit^38^ (χ^2^_df = 376_ = 775.587, CFI = 0.982, TLI = 0.980, RMSEA = 0.041, 90CI [0.037, 0.045], and SRMR = 0.057). The MGCFA yielded two important results: first, the hypothesized model was successfully replicated, and second, structural invariance was present as the items in both the Spanish and Peruvian samples loaded significantly on the latent variables as predicted. Additionally, the two factors had a significant correlation in both groups (r_peruvian_ = 0.55, p < 0.001; r_spanish_ = 0.73, p < 0.001; Fig. 2).

Next, we examined metric invariance by constraining the factor loadings to be equal in both samples. Also, this model showed an adequate fit to the data (χ^2^_df = 395_ = 1014.887, CFI = 0.972, TLI = 0.970, RMSEA = 0.05, 90CI [0.046, 0.053], and SRMR = 0.065). As described above, the measurement invariance involves nested models, which are examined using ANOVA and alternative model fit indices, revealing that the metric model had a significantly poorer fit than the configural model (χ^2^_df = 19_ = 54.197, p < 0.001).

It is important to note that the χ^2^ difference test is sensitive to large sample sizes, which can lead to the quick rejection of models even if they fit the data well. Therefore, we also considered the increase and decrease of AFI. Our findings demonstrated that the metric model was not inferior to the configural model, as shown in Table 3, which supports the assumption of metric invariance. Additionally, we examined scalar invariance by constraining the factor loadings and intercepts to be equal across both samples. The MGCFA yielded a good model fit (χ^2^_df = 414_ = 1270.975, CFI = 0.961, TLI = 0.961, RMSEA = 0.057, 90CI [0.054, 0.061], and SRMR = 0.071). A comparison of both models indicated that the scalar invariance model was significantly poorer than the metric (χ^2^_df = 19_ = 355.91, p < 0.001). However, the change in AFI indicated that the overall fit was not significantly different between the scalar and the metric model (see Table 3), thus supporting the idea of scalar invariance.

**Table 3:** Nested model comparisons: difference between alternative fit indices.

	Δχ2	ΔCFI	ΔTLI	ΔRMSEA	ΔSRMR
Configural vs. metric invariance	239.30	−0.010	−0.010	0.009	0.008
Metric vs. scale invariance	265.10	−0.011	−0.009	0.007	0.006

*χ*^*2*^ Chi-square, *CFI* comparative fit index, *TLI* Tucker–Lewis index, *RMSEA* root-mean-square-error of approximation, *SRMR* root-mean-square-residual.

### Exploratory analysis—differences in CPDI factor scores among groups and prevalent conditions

Firstly, we examined the differences between the Peruvian and Spanish samples regarding stress (factor 1) and rumination/search for information (factor 2) scores. We found no significant differences in either factor 1 (U = 763,562.00, p = 0.167) or factor 2 (U = 780,812.00, p = 0.660).

Furthermore, we investigated the differences in factorial scores among the most prevalent pathologies, including anxious-depressive disorders, respiratory diseases, and hypertension, as listed in Table 1. To identify any differences, we used the U-Mann–Whitney test and excluded participants with multiple pathologies, only including those with the specific pathology under analysis. Factor scores were obtained by correcting items for age, sex, and education level.

**Figure 3:**
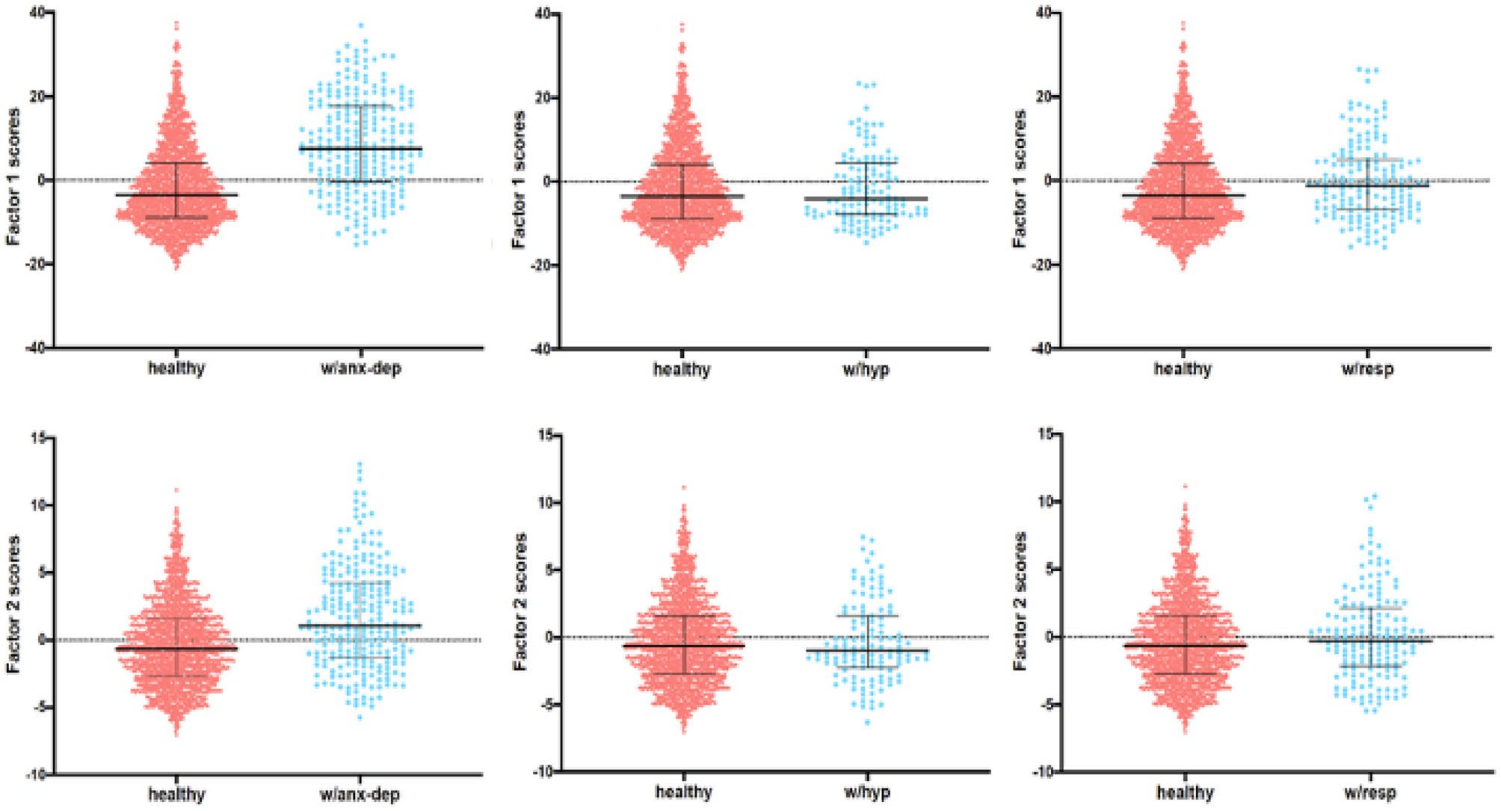
Violin plot charts for CPDI factors 1 and 2 scores in the three most frequent medical conditions of both Spanish and Peruvian samples (*N* = 2543). The median with the interquartile range (IQR) is presented. *w/anx-dep* participants with anxiety-depressive disorder, *w/hyp* participants with arterial hypertension, *w/resp* participants with respiratory diseases. The sum of factor scores 1 and 2 are corrected for age, sex, and educational level (residuals).

The analysis revealed that individuals with respiratory diseases had significantly higher Factor 1 scores than those without (U = 116,697, p = 0.012). However, no significant differences were observed for Factor 2 in participants with respiratory diseases (U = 122,934.50, p = 0.121). Individuals with hypertension showed no significant differences in either Factor 1 (U = 99,412.50, p = 0.638) or Factor 2 (U = 101,534.50, p = 0.928) compared to those without hypertension. Finally, individuals with anxious-depressive pathology had significantly higher scores in both Factor 1 and Factor 2 than those without anxious-depressive pathology (Factor 1: U = 100,454.50, p < 0.001; Factor 2: U = 140,850.50, p < 0.001). These results were independent of age, sex, and education level, as confirmed by the correlation analysis of residuals and represented in Fig. 3.

## Discussion

The results of the present study indicated a two-factor solution for both samples, resulting in a 21-item CPDI instrument. The items of factor 1 were theoretically related to stress reactions in the context of COVID-19, while the items of factor 2 were related to rumination behavior, including the excessive seeking of information in the context of COVID-19. Subsequently, the two-factor solution for the CPDI was confirmed, and it was additionally demonstrated that the CPDI constructs were being measured consistently across both populations (i.e., Spanish and Peruvian). This demonstrates that CPDI can be used in different cultural contexts, confirming the cultural validity of our CPDI model. Finally, our exploratory results showed increased scores for factor 1 (stress reactions in the context of COVID-19) for respiratory diseases and increased scores for both factors in individuals with anxious-depressive disorders. All analyses were adjusted for participant sex, age, and education level.

Previous independent studies have demonstrated a consistent two-factor solution for the COVID-19 Peritraumatic Distress Index (CPDI). In the Spanish study, a two-factor solution was identified through principal axis analysis and *varimax* rotation, which yielded two factors: "*stress symptoms*" (15 items) and "*COVID-19 information*" (8 items). Item 5 was eliminated due to its low factor loading^11^. Similarly, in the Peruvian study, a two-factor solution was found: "*stress in the context of COVID-19*" (13 items) and "*rumination in the context of COVID-19*" (8 items). However, since a correlation between the two factors was assumed, a principal component analysis with a *promax* rotation was performed in this study. Items 7, 8, and 11 were removed from the analysis^14^. When the datasets were combined and analyzed, the same number of factors and a similar distribution of items per factor were obtained (see Table S1). In contrast, other studies^39,40^ used the original four-factor structure of the CPDI, which did not significantly fit our datasets or explain a significant percentage of the variance in our case.

In the second phase, the two-factor model was successfully replicated, and scalar measurement invariance was observed across the Peruvian and Spanish samples. The measurement invariance, as previously defined^41^, demonstrated the psychometric equivalence of the CPDI construct across both groups in the context of COVID-19. This finding indicates that the construct has the same meaning, regardless of the cultural context and variations in the Spanish language (European vs. Latin-American Spanish). Measurement invariance is crucial in scale construction and validation across different cultural and ethnic contexts^42^. For instance, a Canadian study examining the cross-cultural evaluation of the Beck Depression Inventory-II (BDI-II) used MGCFA to demonstrate that BDI-II had measurement invariance across culture and gender^43^. Similar methodologies were used in other psychometric evaluations of psychiatric disorders, such as social phobia^44^, schizotypal personality^45^, and anxiety^46^. The “fear of COVID-19 scale”, which is another evaluation tool to measure psychological distress in the context of COVID-19, was demonstrated to have partial scalar invariance in seven different Latin American countries^47^ as well as in 47 other countries worldwide, demonstrating a scalar invariance across groups concerning culture, gender, and education^15^. Regarding the CPDI, although descriptive and correlational data have been used to establish cross-cultural validity between German and Chinese data^10^, to date, no studies have used the MGCFA methodology to perform cross-cultural validation for CPDI similar to ours.

Our exploratory analysis observed that individuals with respiratory diseases, mainly bronchial asthma, during the COVID-19 pandemic experienced higher psychological stress levels than healthy individuals. Several studies have reported a similar impact of negative emotions during COVID-19 on individuals with asthma. For example, Sheha et al. reported a high correlation between anxiety and depression symptoms and uncontrolled asthma in patients during the COVID-19 lockdown^48^. Similarly, de Boer et al. found a clinically significant increase in anxiety and depression among asthma patients during the pandemic, consistent with our findings^49^. Interestingly, Takeuchi et al. reported that participants with respiratory diseases, such as asthma, pneumonia, and COPD, experienced higher levels of psychological distress than those with cardiovascular diseases or cancer, which is also in line with our results^50^. Regarding anxious-depressive disorders, our findings suggest that participants with these conditions experience higher stress levels and engage in more rumination and information-seeking behaviors compared to healthy individuals. Specifically, rumination, a coping mechanism characterized by negative affect and self-focused attention, appears to be triggered by the repercussions and consequences of the COVID-19 pandemic^51^. This rumination manifests as a constant search for information, which can exacerbate negative and catastrophic thoughts, leading to a feedback loop of repetitive negative thinking^51^. Thus, individuals with anxious-depressive syndromes are more likely to engage in information-seeking behaviors and experience heightened levels of rumination due to the cognitive distortions associated with anxiety and depression, which are also associated with negative behaviors such as seeking out negative news and information^52^.

### Limitations and implications for further research

The present study demonstrates that the Spanish version of the CPDI has consistent psychometric properties in both Peruvian and Spanish samples. While this information is a valuable contribution to the research on psychological stress during the COVID-19 pandemic, some limitations must be considered. Although the sample size of our study was large, the samples from both databases were collected using a snowball method and were not randomized at the time of sampling. Furthermore, most participants were female, well-educated, and between 35 and 50 years old. Additionally, the participants self-reported pathologies, which may introduce memory bias, and were not confirmed by a physician. According to Table S1, the factorial structure of the CPDI does not form clear and stable factors over time, resulting in minimal variations among the excluded questions and suggesting possible heterogeneity in the factors that must be considered. Items measuring psychological distress in the CPDI were non-invariant, as shown in Table S1, including those in the rumination/seeking for information factor. An additional limitation is the lack of testing for convergent and divergent validity. While the sample encompassed two distinct populations and the secondary data analysis demonstrated scalar invariance in the MGCFA, it is important to acknowledge that certain items may exhibit individual loading variations between these groups. These differences could potentially be attributed to socioeconomic factors (e.g., income levels or access to healthcare), sample-specific characteristics, or even response bias, where respondents might provide inaccurate or misleading answers to specific items. In addition, a higher correlation between the two factors was found in the Spanish than in the Peruvian population. In Spain, dealing with news about COVID-19 was more strongly associated with stress perception than in Peru. These results could be interpreted as follows: it might be possible that the Peruvian population managed to deal with negative information about COVID-19 more easily than the Spanish population. Unfortunately, no clear statement can be made about the direction of the effect (between stress and information seeking/rumination), since only correlative data is available. It is crucial to recognize this as a limitation of the study. Finally, longitudinal data may provide stronger evidence for the relation between the two factors of the CPDI and furthermore the factorial structure than a cross-sectional design.

Concerning implications for future research, these should consider applying and validating the CPDI instrument using EFA and perform cross-cultural validations using MGCFA in languages other than Spanish. Moreover, future research should explore whether the CPDI is theoretically related to other scales measuring stressful experiences during the COVID-19 pandemic, which would provide fruitful information. Finally, future research, especially post-COVID research, should evaluate and examine psychometric aspects of psychological stress, using the CPDI, after the pandemic, since the epidemiological and social conditions due to COVID-19 have been changed.

## Conclusions

In conclusion, this study provides evidence for the cross-cultural validity of the CPDI as a reliable instrument for assessing psychological stress in the context of COVID-19 across different Spanish-speaking cultures. These findings have important implications for developing and validating measures to assess psychological distress in other cultural contexts. Furthermore, our findings carry significant implications for the CPDI as a reliable, culturally robust, and valid instrument for assessing peritraumatic stress. Consequently, it should be regarded in future studies as a valuable tool and adapted in the future for similar events, including potential future pandemics, such as another coronavirus outbreak. Our exploratory analysis revealed that respiratory diseases and anxious-depressive disorders significantly increased psychological stress during the COVID-19 pandemic. The latter also correlated with increased rumination and seeking of information, which is consistent with the psychopathology of both disorders.

### Supplementary Information

Supplementary Table S1.

## Data Availability

The data sets generated and analyzed during the study are not publicly but they are available from the corresponding author on justified request.
