# Supplementary material for: Cross-cultural validation of the COVID-19 peritraumatic distress index (CPDI) among Spanish and Peruvian populations
Source: Sci Rep. 2023 Nov 3;13:19005. doi: 10.1038/s41598-023-46235-4 (PMC10624667; doi:10.1038/s41598-023-46235-4)
Supplement: Supplementary file 1 — Supplementary Table S1. [file 41598_2023_46235_MOESM1_ESM.docx]

**Böttcher F., Pedraz-Petrozzi B, et al. Supplementary material**

|  | **CPDI items** | **Eliminated CPDI items** |
| --- | --- | --- |
| **Spanish study** | **Factor 1 (*stress*):**  1, 4, *7*, 12, 13, 14, 15, 16, 17, 18, 19, 20, 21, 23, 24    **Factor 2 (*information*):**  2, *3*, 6, *8*, 9, 10, *11*, *22* | 5 |
| **Peruvian study** | **Factor 1 (*stress*):**  1, 4, 13, 14, 15, 16, 17, 18, 19, 20, 21, 23, 24    **Factor 2 (*rumination/seeking for information*):**  2, *3*, *5*, 6, 9, 10, 12, *22* | 7,8 and 11 |
| **Combined data** | **Factor 1 (*stress*):**  1, 4, *7*, 12, 13, 14, 15, 16, 17, 18, 19, 20, 21, 23, 24    **Factor 2 (*rumination/seeking for information*):**  2, 6, *8*, 9, 10, *11* | 3, 5 and 22 |

**Table S1** - CPDI items and eliminated items in the Spanish study, Peruvian study, and in the secondary analysis of the combined data. Items that are consistently in the factor are underlined, while the eliminated items in the different studies are written in italics. Item 12 changed factors between the studies.
